# Experimental and Theoretical Model of Single Vessel Minimally Invasive Micro-Laser Ablation: Inducing Microvascular Network Remodeling and Blood Flow Redistribution Without Compromising Host Tissue Function

**DOI:** 10.21203/rs.3.rs-3754775/v1

**Published:** 2023-12-18

**Authors:** Gabriel Gruionu, James Baish, Sean McMahon, David Blauvelt, Lucian G. Gruionu, Mara Onita Lenco, Benjamin J. Vakoc, Timothy P. Padera, Lance L. Munn

**Affiliations:** 1 Indiana University School of Medicine, Krannert Cardiovascular Research Center, Department of Medicine, Indianapolis, 46202, USA.; 2 Massachusetts General Hospital and Harvard Medical School, Edwin L. Steele Laboratory for Tumor Biology, Department of Radiation Oncology, Boston, 02114, USA.; 3 University of Craiova, Department of Mechanical Engineering, Craiova, 200585, Romania.; 4 Bucknell University, Department of Biomedical Engineering, Lewisburg, 17837, USA.; 5 Virginia Tech, Department of Physics, Blacksburg, 24060, USA.; 6 Boston Children’s Hospital, Department of Anesthesia, Critical Care, and Pain Medicine, Boston, 02115, USA.; 7 Trinity Life Sciences, Waltham, 02451, USA.; 8 Harvard Medical School and Massachusetts General Hospital, Department of Dermatology and Wellman Center of Photomedicine, Boston, 02114, USA.

## Abstract

Overly dense microvascular networks are treated by selective reduction of vascular elements. Inappropriate manipulation of microvessels could result in loss of host tissue function or a worsening of the clinical problem. Here, experimental, and computational models were developed to induce blood flow changes via selective artery and vein laser ablation and study the compensatory collateral flow redistribution and vessel diameter remodeling. The microvasculature was imaged non-invasively by bright-field and multi-photon laser microscopy, and Optical Coherence Tomography pre-ablation and up to 30 days post-ablation. A theoretical model of network remodeling was developed to compute blood flow and intravascular pressure and identify vessels most susceptible to changes in flow direction. The skin microvascular remodeling patterns were consistent among the five specimens studied. Significant remodeling occurred at various time points, beginning as early as days 1–3 and continuing beyond day 20. The remodeling patterns included collateral development, venous and arterial reopening, and both outward and inward remodeling, with variations in the time frames for each mouse. In a representative specimen, immediately post-ablation, the average artery and vein diameters increased by 14% and 23%, respectively. At day 20 post-ablation, the maximum increases in arterial and venous diameters were 2.5x and 3.3x, respectively. By day 30, the average artery diameter remained 11% increased whereas the vein diameters returned to near pre-ablation values. Some arteries regenerated across the ablation sites via endothelial cell migration, while veins either reconnected or rerouted flow around the ablation site, likely depending on local pressure driving forces. In the intact network, the theoretical model predicts that the vessels that act as collaterals after flow disruption are those most sensitive to distant changes in pressure. The model results match the post-ablation microvascular remodeling patterns.

## INTRODUCTION

The blood vasculature is topologically organized as branched trees or a hybrid combination of trees and connecting vessels between branches, which effectively create loops^1,3^. Most vessel networks, including the skeletal^4–6^ and cardiac muscle^7–11^ and skin vasculature^12^ exhibit the hybrid topological organization which provides functional advantages while minimizing flow resistance. The vessels that interconnect adjacent branches of the same vascular tree are known as collaterals or arcades, and these provide redundancy and alternate pathways for flow through the network.

Blood vessels respond to alterations in flow in normal and pathological conditions^2,4,13^ by changing both the inner vascular diameter and the vascular wall thickness^2,4,13^. Vasomotion and changes in arterial tone control the overall flow of blood to specific tissues and can also redirect flow to specific regions of the network. Depending on regional demand, the flow in collateral vessels can stagnate or reverse, thus controlling flow laterally across a network rather than down the branching hierarchy. Collateral vessels are generally redundant in baseline tissue function, but are very important during microvascular alterations such as reestablishing blood supply during ischemic revascularization^4,14–17^, or temperature control^18^ and brain function^19,20^.

Selective manipulation of vascular networks can be used in the clinic to enhance microvascular function or reduce vessel density. Diabetic retinopathy (abnormal vascular growth of retinal vasculature) is treated locally by selective laser photocoagulation to reduce vascular density^21,22^. Preclinical studies in other tissue beds (cremaster muscle, mesentery, saphenous and femoral artery and vein) showed significant vascular remodeling following laser ablation^23^, but an integrative and predictive network model which can serve as a planning tool for clinical interventions is still lacking.

**Figure 1.**
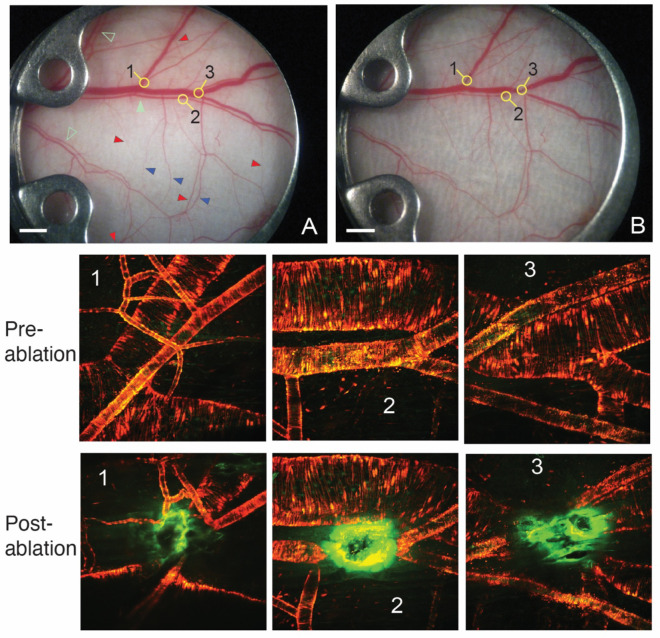
Vascular laser ablation. A, B: Bright field (BF) microscopy images of the skin vasculature before (A) and at four hours after ablation (B) of specific large vessels. The major and minor artery-vein pairs are indicated by solid and open green arrowheads, respectively. The three regions of ablation are shown with the numbers. Red and blue arrowheads indicate some of the major arcading/collateral vessels on the arterial and venous sides, respectively. The lower panels show multiphoton laser microscopy (MPLM) images of microvasculature before and immediately after laser ablation of each region indicated. Red: smooth muscle cells (aSMA-DsRed); green: endothelial cells (TIE-2-GFP); orange: overlapped SMCs and ECs; bright green: autofluorescence of scar tissue resulting from the ablation procedure. White scale bars are 1mm.

In the present study, we examined vascular redundancy and the ability of vascular networks to adjust their diameter and redirect flow following focal ablation of blood flow while the remaining network was undamaged (Figure 1). Our hypothesis was that alterations in blood flow to major vessel segments in a microvascular network should result in predictable remodeling of the remaining vessels, collateral pathway development and redistribution of blood flow to areas with decreased supply. To test this hypothesis, we selectively laser-ablated arterial and venous vessels in the dorsal skin vasculature of five mice and measured the topological changes.

We then analyzed one representative network in more detail to demonstrate how a computational model of microvascular flow distribution might be used to predict which collateral vessels are redundant in a microvascular network and are susceptible to compensatory flow reversal in response to adjacent vessel blockage. The model was used for two related purposes. First, using the available data, we demonstrate simulated annealing as a tool for estimating unknown relative pressures at the edges of the field of view based only on the directions of flow within the observed network. While independent measures of these pressures are currently not feasible, our simulated annealing method provides information about the uncertainty in the predictions. The second use of the model is to demonstrate the concept that the redundant vessels that permit rerouting of flow around a disruption can often be identified as those with flow that is most sensitive to changes in pressure distal from the ablation. A tool for making such predictions is likely to be valuable when planning selective ablations in settings such as the retina or coronary circulation. Similar computational models can be developed to test clinical hypotheses in other microvascular networks.

## RESULTS

### Laser ablation of the dorsal skinfold chamber (DSFC) microcirculation

The general patterns of skin microvascular remodeling were similar in all five mice studied (Supplementary Figure 1, sFig. 1 and Supplementary Tables 1–6, sTables 1–6). sFig.1 provides an extensive depiction of the time course and patterns of microvascular network remodeling observed in five distinct DSFC experiments, denoted as Rows A-E, each row representing a separate animal experiment. For the in-depth analysis, we focused on Mouse E as a representative case. For this network, we performed a comprehensive anatomical data analysis and mathematical modeling. In sFig. 1, the locations of laser ablations are denoted by the circles (red, arterial; blue, venous). White crosses signify collateral outward remodeling from previously very small vessels, and blue crosses represent outward or inward remodeling of existing arterial/venous segments. Red and blue brackets indicate arterial and venous ablated segment reopening, respectively, and red and blue cross-brackets denote interruption of perfusion in arterial and venous segments, respectively. One to three ablations (except for mouse B D1+) were performed at select locations in the middle of the microvascular network in the largest visible arteries and veins. Remarkably, all specimens exhibit substantial remodeling at different time points from as early as days 1–3 (sFig.1, Row B d1 and mouse C d3) and up to day 20 (mouse E, d20) and later (see below). In sFig.2, mouse A, proximal venous ablation was bypassed through the development of an existing transverse venule, which underwent outward remodeling to match the initial vein diameter. The distal venous ablation revascularized by day 12, while the main vein initially underwent inward remodeling until day 12 and subsequently returned to its pre-ablation diameter by day 17. Arterial ablations and one venous ablation reopened by day 12 in mouse A. In mouse B, by day 5, venous ablations either led to bypass through outward remodeling of transverse veins (mouse C, d5, upper half) or caused inward remodeling of the main venous branch (mouse C, d5, lower half). Mouse C illustrates venous ablations bypassed by pronounced collateral development, while arterial ablation successfully revascularized. In mouse D, initial arterial and venous ablations reopened as early as day 2, while other ablations targeted the main artery and vein and two of their branches to induce more permanent flow changes. Mouse E showcases a combination of all remodeling patterns, albeit with varying time courses. Venous ablations revascularize through collateral growth, and arterial occlusions reopen. The majority of vessels display visible remodeling, and diameter data is further described and modeled in subsequent sections of the study.

The detailed diameter values are reported in sTable 1 for intact pre-ablation vessels and sTables 2–5 for remodeling time points reported in sFig.1 for the proximal, medial, and distal regions from the closest ablation.

The primary observed remodeling patterns, which encompass outward/inward remodeling of existing arteries and veins, collateral growth of previously small vascular segments, segment reopening, and permanent segment occlusions, are summarized in sTable 6, with accompanying diameter data provided in sTables 2–5, and illustrated in sFig. 1. One of the notable findings was the presence of both outward and inward remodeling phenomena in both arterial and venous segments, a dynamic process that persisted throughout the observation period. From the onset, the immediate aftermath of laser ablation at day 0 there were significant diameter changes as shown in sTables 2–5 although these changes are difficult to observe in sFig. 1. Starting at day1, there was visible collateral remodeling in most specimens.

Furthermore, sTable 6 also highlights the segment occlusion which was the goal of each initial laser ablation. While certain vessels maintained their occluded state throughout the observation period, a subset of vessels displayed the ability to gradually reopen over time. This observation indicates the dynamic nature of microvascular responses and their potential for adaptive adjustments over extended timeframes.

Due to variations in time course remodeling among specimens, a representative mouse (mouse E in sFig. 1 and sTables 1–6) was chosen to show the observed remodeling process for the remainder of the study. The typical mouse microcirculation within the DSFC contains a main artery and vein pair (Fig. 1A, B, solid green arrowhead, and sFig. 1) and smaller artery - vein pairs (open green arrowheads). There are multiple arcade/collateral vessels that connect arteries to other arteries on separate branches of the arterial or tree or veins to veins between venous branches. A few arterial collaterals are indicated by red and venous collaterals by blue arrowheads, respectively in (A). These arcading vessels provide vascular redundancy by allowing redistribution of blood flow. Arteries have significantly smaller diameters than the paired veins and have tighter concentric layers of smooth muscle cells (red and yellow in Fig. 1, Pre-ablation 1–3 and Post-ablation 1–3).

The laser ablation was performed at three major locations (Fig. 1A and B, two artery/vein pairs in regions 1 and 3, and an artery in region 2) in the center of the window to maximize blood flow redistribution and to allow long term observation of the developing vascular changes (as some drifting of the tissue occurs within the DSFC over two weeks). The ablated vessels experienced rapid vasoconstriction upstream and downstream from the ablation site (Fig. 1, Post-ablation 1–3). There was complete blood flow interruption in segments just distal and proximal from the ablations (sVideos 1A, 2A and 3A). The laser ablation procedure was focused only on the target vessels, effectively cauterizing them while having little effect on the surrounding tissue. The brown scar tissue located in the muscle fascia subsides at later time points (sVideos 1B, 2B and 3B). Note that in region 2, the ablation of the artery had no effect on the diameter of the adjacent large vein or the blood flow in that vessel (Fig. 1, Post-ablation 2; sVideo 2A).

### Time-course of vascular network remodeling

**Figure 2.**
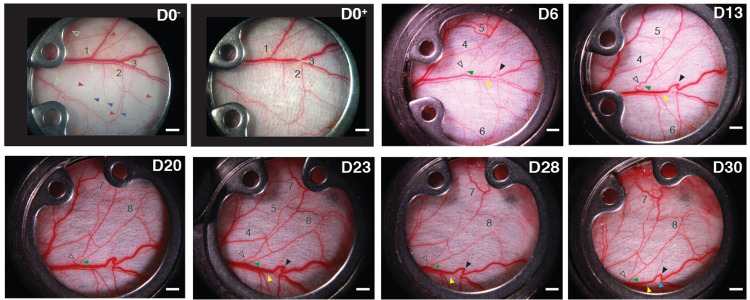
Time course of vascular remodeling post-ablation. D0^−^ and D0^+^ indicate pre-ablation and post-ablation on Day 0, respectively. Regions 1–3 indicate the ablation regions and site (yellow line). Shown are images through Day 30 (D30). Initially, at Days 6 vessel redundancy and remodeling in areas compensate for the ablation-induced ischemia. By day 13, the venous connections were reestablished (clear and black arrowheads). From day 20, the artery in Region 1 has reconnected (green arrowhead), increasing flow to the downstream network. There was no angiogenic regeneration of the ablated veins; instead, flow quickly rerouted through small pre-existing venules that appeared to pre-existing connections at either side of the damage site (clear and black arrowheads, D13–30). The white scale bars are 1mm.

By day 6 after ablation, there was clear evidence of vascular remodeling throughout the network (compare Figs. 2D0−/+ and 2D6). Vessel segments associated with the ablated vessels had reduced diameter at day 6, while there was increased diameter in a number of collateral vessels (regions 4, 5 in Fig. 2D6). By day 13, vessel diameters had qualitatively returned to pre-ablation values for much of the network (Fig. 2D13). This was due to remodeling of collateral vessels, which allowed an increase in compensatory flow entering tissue regions previously supplied by the ablated vessels. There were also large increases in diameter in a few small vessels that restored flow through the veins by bypassing the ablation sites (arrowheads in Fig. 2, D13, and sVideo 1A-D, 3A-D).

These structures formed from sequences of smaller microvessels that were part of the original vascular bed. The increased flow through these small bypass channels likely caused the expansion of vessel diameter which eventually matched that of the original vein, similar to previous observations in the mouse gracilis muscle^2^.

Some branches from the two small networks in regions 1 and 3 associated with the new vein segments appeared to be pruned or regressed as the new segments became part of the large veins. Albeit observed at low/medium resolution in transmitted light images, in these veins, there was no visible evidence of extensive angiogenesis or new vessel growth contributing to the regeneration of the network or restoration of flow. Rather, the rerouting occurred through remodeling of existing vessel segments, most of which could be visualized even before the ablations were performed (see Fig 5a, b).

However, we did observe reconnection of venous segments through the ablation site via endothelial migration in other networks (sFig.1, rows A-C and E). The response to injury appears to be related to the effective blood pressure difference across the ablation. In Fig. 2, regions 1 and 3, the ablations are situated such that there is a large pressure drop across the ablation sites. This forces the blood to reroute through the smaller vessels early after the injury. However, in sFig.1 row A, there were two ablations performed on the same large vein. In this case, the upstream ablation has little pressure drop because the downstream ablation is preventing outflow. For this reason, very little flow re-routing or vessel remodeling occur at the upstream ablation, and this region was instead reperfused by direct reconnection of the vein via angiogenesis (sFig. 1, row A, d12 and d17).

On the arterial side, in region 2 we did not observe re-routing locally through pre-existing microvessels, and their subsequent enlargement, as in the veins of regions 1 and 3, Fig. 2. Instead, flow was redistributed through the preexisting arterial arcades to circumvent the ablation and compensate for the lowered flow distal to the ablation sites (Fig. 2. D6 and D13, areas 4–6, and sFig. 1C, d3 and d18). Compared with the venous rerouting in regions 1 and 3 in Fig. 2, which occurred over very short distances (~1mm) around the ablations, rerouting on the arterial side extended over much larger distances (~5–10mm) through the arcade vessels. In the ablated arteries, we did observe reconnection of the vessel through the ablation site via angiogenesis. On days 20, 23, 28 and 30, there was evidence of regeneration on the arterial side, as the artery ablated in Region 1 (Fig. 2) reconnected (for example, see the arterial ablation in region 1 (Fig. 2, D6–30, green arrowheads, and sVideo 1A-D). As this new vessel segment grew, original flow through the artery was restored, and the diameters of the major compensating collaterals decreased (Fig. 2, D28, region 8). The artery in region 2 (Fig. 2, D6–30, yellow arrowheads) did not achieve reconnection by the 30-day time point although some small flow pathways can be traced (sVideos 2C and 3C). The arterial flow in region 3 was re-established by day 30 but via smaller vessels than the original artery (Fig. 2, D30 blue arrowhead), with blood flow evident via doppler OCT at day 14 (Fig. 5, D14b) and intravital BF imaging at later time points (sVideo 3D).

### Angiogenesis at the ablation sites

**Figure 3.**
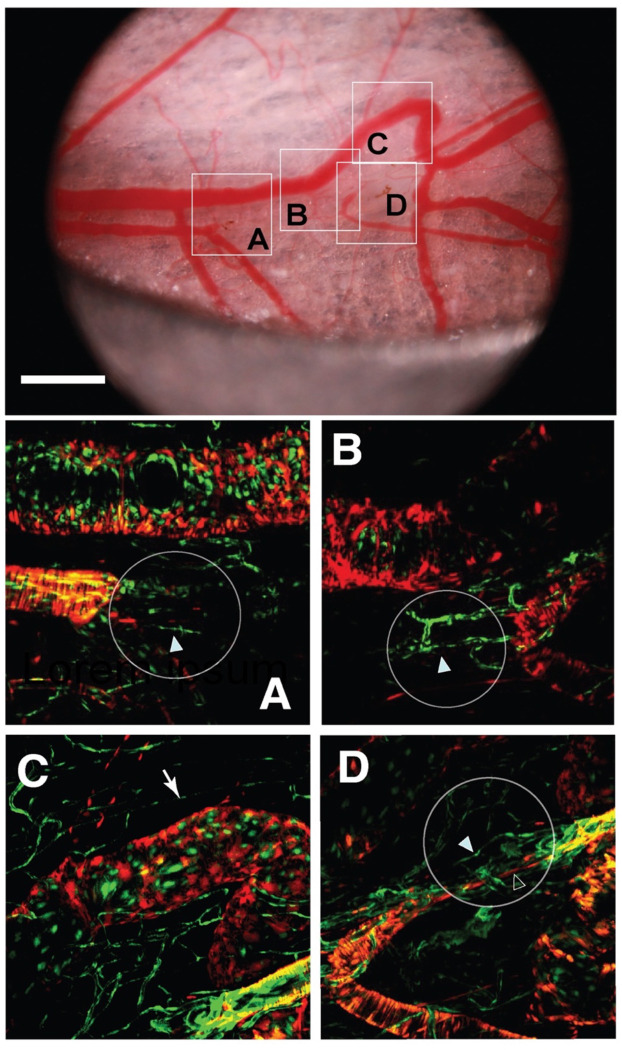
Vessel regeneration at Day 30. At top is a brightfield image of regions 2 and 3 from Figure 1. Four regions are shown in detail with multiphoton imaging of the endogenous TIE2-GFP (endothelial cells) and aSMA-dsRed (smooth muscle cells). The ablated regions are shown by the circles. In these regions, there was evidence of angiogenesis in the arterial network as endothelial cells (solid arrowheads) and smooth muscle cells (open arrowhead) migrated into the ablated regions. At this time point, the remodeled vein segment in region 3, Fig. 1 has matured, with a covering of smooth muscle cells (arrow, C). The scale bar is 1mm.

Because of the endogenous reporters expressed by the mice, we were able to visualize endothelial cells (TIE2-GFP - green) and smooth muscle cells (aSMA-dsRed - red) longitudinally at the ablation sites. *In vivo* laser confocal imaging of the regions 2 and 3 in Figure 1 revealed migration of the endothelial and smooth muscle cells through the ablation sites (Fig. 3). In region 3, the vascular pathway was re-established, and blood flow was observed (Fig 3D). Both endothelial and smooth muscle cells migrated into the damaged region and appeared to establish a connection by day 30, based on doppler OCT imaging (see Figure 5). A similar process was observed for the other artery, which was ablated at location 2 in Figure 1 (Fig. 3A, B), although this vessel did not reconnect by the end of our observation period. Angiogenesis was not observed in the large vein that remodeled in region 3, but the remodeled region acquired a covering of smooth muscle cells (Fig. 3C). After day 30, the relevant vessels had shifted out of the window chamber and were no longer observable.

### Time course of diameter remodeling

**Figure 4.**
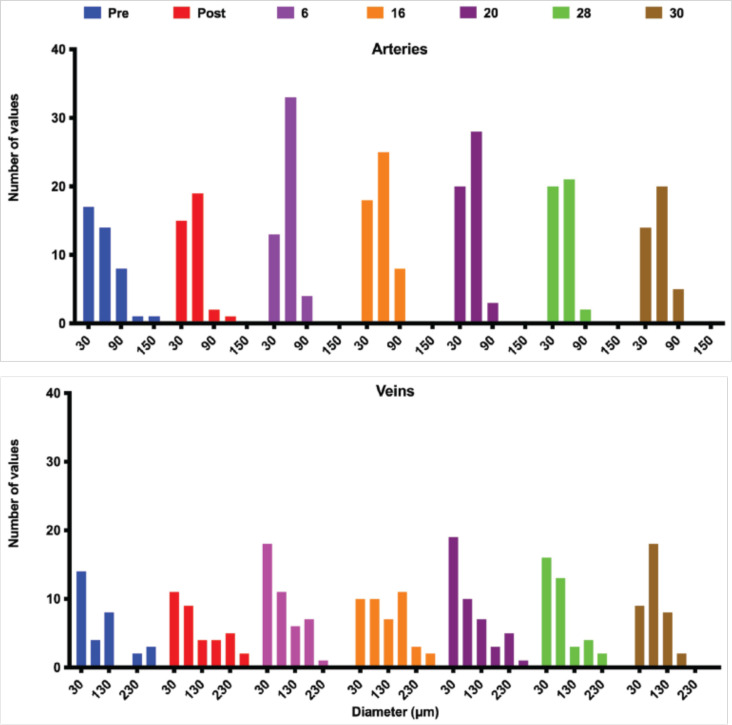
The frequency distribution of vessel diameter for arteries (top) and veins (bottom) pre and up to 30 days post-ablation. Post-ablation, the distribution of artery diameters is skewed towards more smaller diameter vessels suggesting the blood is redirected from large arteries to smaller alternative pathways. This trend is reversed towards a more normal distribution (more larger vessels) past day 16. On the venous side, the distribution of diameters is more stable reflecting a larger capacity of the venous side to accommodate blood flow redistribution without major diameter changes in most of the vessels.

Overall, both arteries and veins changed their diameters collectively over time (Fig. 4 and sFig. 1 and sTables 2–6). Because of resolution limitations, we restricted the quantitative analysis to the main arteries and veins and their transverse branches with inner diameters larger than 11 μm; therefore, the histograms do not include smaller vessels and capillaries. The smallest arteries (30 μm centered bin) stayed almost constant during the time points studied. A small dip at day 6 was recovered and slightly increased at the later time points. Combined with changes at other time points this could mean that smaller vessels became larger and therefore visible in this diameter range. The largest change in diameter distribution was observed in the 60 μm bin which was increased at days 6–20 and went back to normal values by day 30 which suggests a transient increase in vessel diameters to accommodate the early changes in blood flow as we noticed before in the gracilis artery remodeling^2,4^. Some larger vessels also constricted, moving from the 90–150 μm to the 60 μm range. At day 16, this trend reversed temporarily while between days 20–28 a lot of the larger arteries were still constricted. By day 30 diameter distribution of all arteries was close to post-ablation and pre-ablation values even in with the absence of the ablated large artery suggesting that blood redistribution can be accomplished through the contribution of the network of smaller arterioles even in the absence of the large artery.

The vein diameter distribution is more spread over a larger range of diameters suggesting a larger adaptation of the veins to accommodate flow changes. The largest variation in diameter distribution was observed in the 30 μm bin although slight transient tendency is also observed between days 6 and 28 with a decrease to normal values at day 30. During the transient increase period, an interesting second transient decrease was observed at day 16. Veins in the 80 μm range exhibited a gradual increase starting from post-ablation and peaching at day 30. The veins with diameters in 130–180 μm range showed the larges increased in density at early and medium time points (days 6 and 16). The largest veins stayed open immediately following the ablation, at day 6 they were reduced in diameter, at days 16 and 20 they were close to normal values but by day 30, the number of larger veins was drastically reduced suggesting again that on the venous side like the arterial side, flow redistribution could also be accomplished via a larger network of smaller venules.

**Figure 5.**
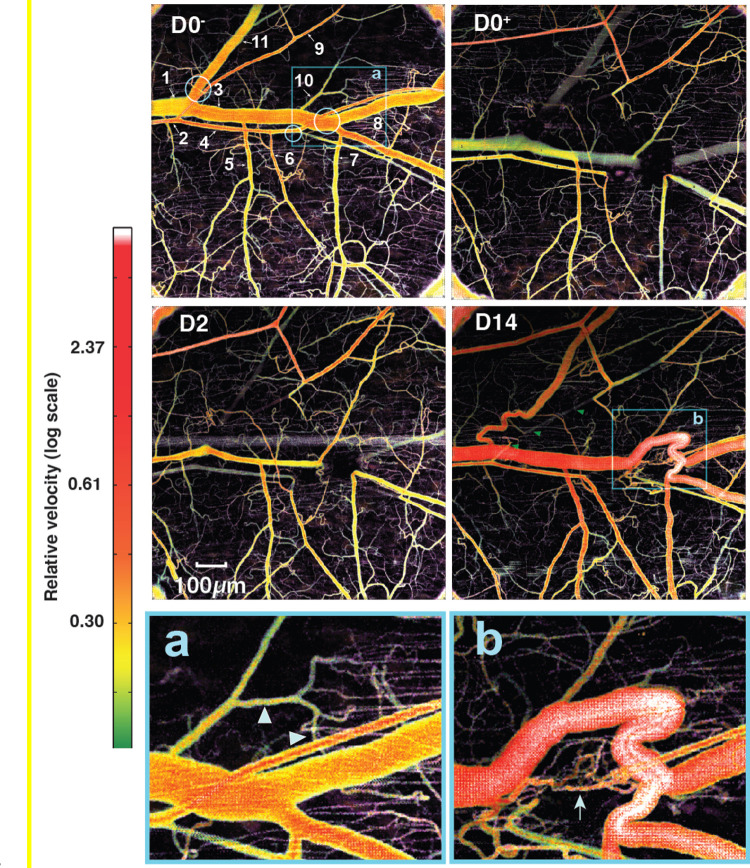
Blood flow visualized by decorrelation-based quantitative flowmetry OCT before ablation (D0^−^), just after ablation (D0^+^) and on days 2 (D2) and 14 (D14). The three ablation sites are marked with blue circles at D0^−^ (see also Figs. 2 D0^−^ and D0^+^). Areas in the blue boxes at D0− and D14 (a, b) appear at bottom at higher magnification. Immediately post-ablation, flow is completely interrupted in the segments just downstream from the ablations and diverted to alternative pathways. The venous connection in left side ablation site (circle 1 in Figs. 2 D0^−^ and D0^+^) is reconnected by day 14 while the arterial segment is not reconstructed. The flow is reversed in artery 6 which received blood from the bottom vascular network from day 0 to day 30 when the direction of flow is restored to pre-ablation direction from the large artery segments 2 and 4 towards segment 6 (Supplementary Videos 1–3). Venous segment 10 remodels close to 400% from a venule to a major vein. Smaller post-capillary venules also appear to be involved in this rerouting of flow (arrowheads). By day 14, angiogenesis has partially reconnected the artery in this region, and some flow is evident (arrow, b).

We next focused on individual vessels to determine how specific vessels contributed to the flow redistribution. Using quantitative flowmetry OCT methods based on amplitude-decorrelation which can be used to estimate flow rate as well as lumen diameters ^36,37^, we analyzed a number of segments distal and proximal to the ablations sites before and following the ablations (Fig. 5). We also used intravital BF microscopy to determine flow directions (see Supplementary Videos 1–3). In the intact network, the blood flows from left to right from the large artery (#2, Fig. 5) to its branches (#4, 6 and 9). The blood flows from the venous branches (#3, 5, 7, 8 and 10) towards the main vein (#1). Following ablation, the blood flow stopped in the ablated segments, but both upstream and downstream arteries continued to be perfused by arcading vessels from adjacent vascular trees (#2,4,6 and 9). Immediately after and at day 2 post-ablation, the segments near the ablations were not perfused. Nonetheless, at day 14, there is a signal of blood flow (Fig. 5, D14 green arrowheads) confirming the data from bright field microscopy (green arrowheads in Fig. 2, D6–30). The arteries upstream from the ablation (#2 and 4) have a decreased diameter and flow velocity during the first few days post-ablation while the more peripheral arteries (#6 and 9 with reversed flow as observed experimentally) increased their diameters from day 2 post-ablation and through day 14, suggesting that they are largely responsible for the compensatory flow being rerouted from the parallel arteries (which are outside of the field of the window).

The main vein (#1 and 3) significantly decreased its diameter at day 2 but by day 14 the main vein and its small branch (#10) as well as a contiguous series of microvessels became enlarged to match the size of the vein (Fig 5a, b). Venous branch #5 maintained its diameter throughout the 14-day time-course, as its flow was not directly affected by the ablations, and exit flow proceeded through the main vein through this pathway. After the ablation, flow through vein #7 was rerouted through vein #8, causing flow reversal in this vessel (Supplemental video 3A). Once the connection between these segments and the main vein was reestablished, the flow direction in vein #8 returned to normal (Supplementary video 3B). These changes in flow direction and topology resulted in large changes in diameter and flow rate in this region (Fig 5, D14). A side branch, venule #10 was affected little by the ablations, and maintained exit flow through the main vein. The ablation completely stopped exit flow in vein #11 by day 14, the connection is rerouted, and flow and diameter are returning to pre-ablation levels.

**Figure 6.**
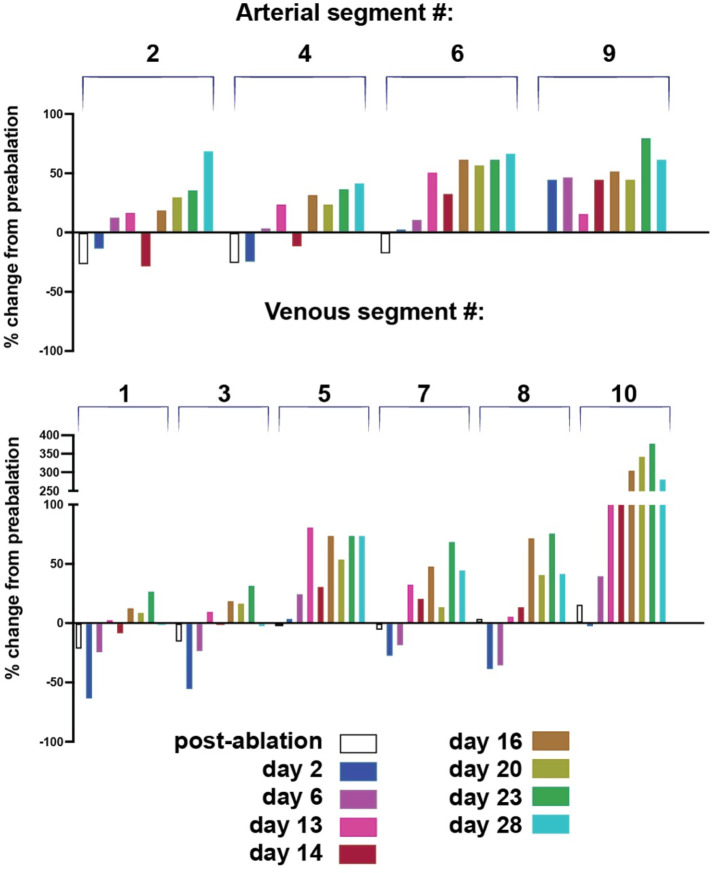
Time course of diameter changes for the representative vessel segments imaged by OCT (see Fig. 5). The venous connection in area 1 is re-established by day 14 while the arterial segment #2 is not reconstructed. The flow is reversed in artery 6 which received blood from the vessels of the distal network at the bottom region of the Figs. 1 and 2. Venous segment 10 remodels close to 400% from a precapillary venule to a major vein.

Diameter measurements at later time points show that main artery segments #2 and 4 recover after the initial diameter decrease probably due to vasoconstriction caused by the ablation. They continue to remodel outwards from day 16–28 with a transient dip at day 14 (Fig. 6 top histograms). The transverse arteriole #6 diameter increased throughout the time course although the flow direction changed (Supplementary videos 2A-C). Despite interruption from the main artery 52, its distal arteriole branch #9 had undergone outward remodeling (with a transient lower rate at day 13) due to collateral and reversed flow from adjacent arterioles.

The main vein segments #1, 3 and 8 remodeled inward at early time points and then outward from day 14 on. The transverse venules #5 and 7 remodeled outward, likely to compensate for the main vein interruption. Interestingly, the distal part of the small venule #10 remodeled outward rapidly to match diameter and re-route flow to the main vein. Its diameter increased by 40% at day 6 to 221% at day 13, 229% at day 14, 306% at day 16 and 343% at day 20. Vessel #10’s outward diameter remodeling peaked at day 23 at 379% increase from normal (close to 400%) and decreased by the end of the observation period at day 28 to 282% of the original diameter at day 23, suggesting a possible transient remodeling (Figure 5b and Fig. 6, venous segment #10).

### Computational model simulation

**Figure 7.**
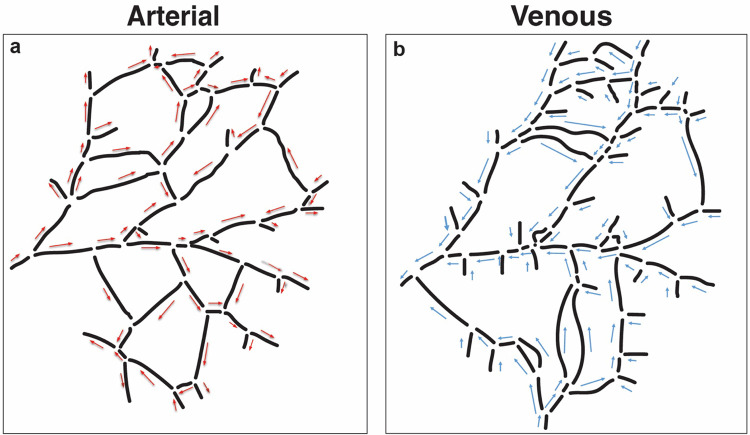
Vascular network topology and flow patterns. The arterial (**a**) and venous networks (**b**) are traced separately based on intravital images, and digitized versions are extracted. The observed flow directions are indicated by arrows.

We next investigated flow patterns in the network before and after the ablations. To do this, we used a computational approach to estimate flow in each segment. The first step in computational modeling is extraction of the network topology and characterization from bright field images taken with the stereo microscope (Fig. 7). The venous network roughly parallels the arterial network with visibly larger diameter vessels. The direction of the flow for each segment was observed from the live BF microscopy recordings and marked on the network map (Fig. 7a and b).

**Figure 8.**
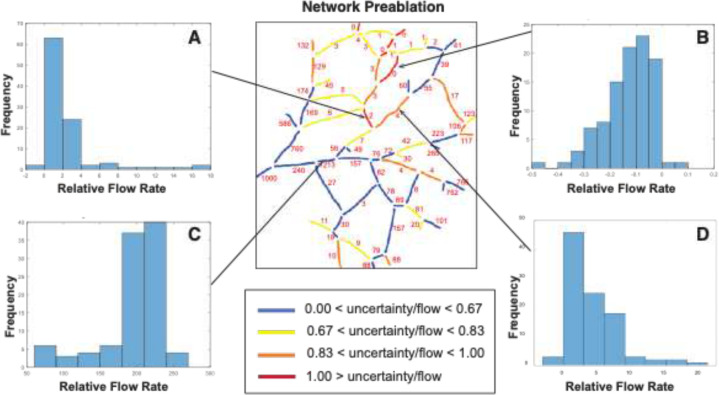
Computational model results of the pre-ablation network. Flow rates have been normalized relative to a value of 1000 assigned to largest vessel segment located on the left side. A-D) The histograms show the frequency of flow rates in representative vessel segments obtained from 100 runs of the simulated annealing algorithm. Numbers in the network map show the average flow rate for each segment, calculated over the 100 runs. The network map is color coded to show the relative uncertainty (standard deviation/mean) of the flow rates in each segment.

We then used a simulated annealing method to estimate flow rates and pressures throughout the network (see Methods). Guesses are made for the terminal segment pressures, and the flows are calculated based on topology and measured vessel diameters. The predicted flow direction in each segment is compared to the observed direction, and an error function is calculated based on the number of incorrect directions. The error is used to scale a set of new guesses for the pressures, which is also subjected to a random function (this is the basis for the simulated annealing method). The process is then repeated to minimize the number of incorrect flow directions in individual segments. Using this method, we find that most large vessels have flow that varies little between trials (blue in Fig. 8), but that flow direction in a few vessels (red in Fig. 8) is relatively uncertain - showing a high sensitivity to distant changes in pressure. This suggests that these vessels can readily serve as collaterals that are available to redirect flow in either direction if necessary.

First, the flow distribution of individual vessels was optimized based on network topology and flow directions in the normal non-ablated state for vessels with different levels of uncertainty/flow levels (Fig. 8). Before ablation, the larger arteries have low uncertainty, suggesting that they rarely change flow direction (Fig. 8, blue and yellow color vessels). For example, the vessel fragment in Fig, 8, panel C has a low level of uncertainty (indicated by blue color on the vessel map) and the relative values of the volumetric flow rate are mostly around 20% of that in the largest vessel (which is assumed at a value of 1000). The segments with the highest uncertainty mostly carry lower flow and are located near the center of the network (Fig. 8, red and orange color vessels). To illustrate this point, vessel fragments in Fig. 8, panels A, B and D have a higher uncertainty (orange and red on the vessel map) and therefore a wider range of possible values. Note that the segments in panels A and B stabilize at zero or close to zero values which reflects a low priority for these collateral vessels prior to ablation.

**Figure 9.**
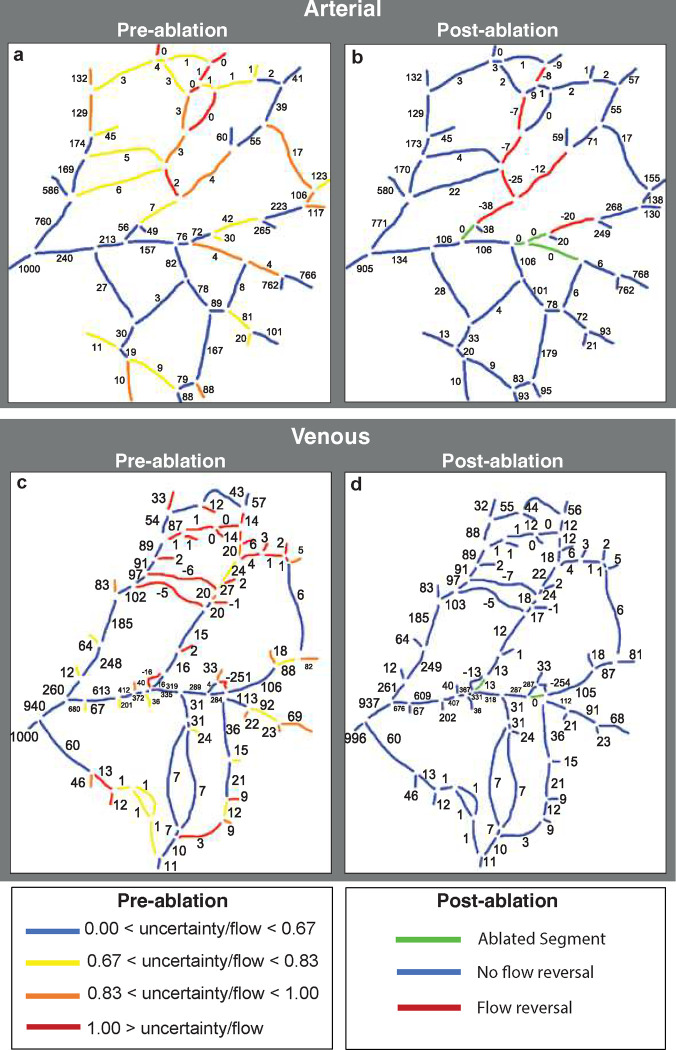
Computational model results of the pre- (a and c) and post-ablation (b and d) networks. Numbers in the network maps indicate flow rate. The arterial network (a and b) had fewer fragments with high flow rate uncertainty than the venous network (c and d). However, flow reversal was common in the arteries but not the veins.

Using this method, we estimated flow through the network before (Fig. 9 A and C) and after ablation (Fig. 9, B and D) for arteries and veins, respectively. The venous network had more segments with higher flow rate pre-ablation (Fig. 9, C vs. A). In arteries, after ablation, flow tends to be reversed in vessels with a high uncertainty index in the pre-ablation model close to the site of ablation (Fig. 9). There was no flow reversal in the vein network although the flow magnitude was slightly changed in many vessel fragments.

## DISCUSSION

In this study, we analyzed flow redistribution and remodeling in vessel networks after laser ablations of vascular segments. Laser ablation cauterizes vessels and causes acute temporary vasoconstriction lasting only a few minutes after ablation. This was observed in the ablated vessels as well as segments downstream and upstream from the ablation location. The diameters of the vasoconstricted vessels returned nearly to control values within a few minutes of ablation. This is direct evidence that the vasoactivity of the ablated vessels and their immediate branches was not altered by the laser ablation. This new approach can be further used to investigate shear-based remodeling in injured or developing networks.

The specific diameter changes and patterns of remodeling were observed in detail in five specimens. The present data demonstrated that microvascular remodeling patterns are similar and reproducible but differ in detail from mouse to mouse (sFig. 1 ad Tables 2–6). The comprehensive data presented in sTable 6 not only underscores the diversity of remodeling patterns but also the intricate and adaptive nature of microvascular networks in response to laser ablation, offering valuable insights into their behavior and potential clinical relevance.

sFig. 1, in conjunction with sTables 2–6, provides a comprehensive insight into the dynamic behavior of microvascular networks in response to laser ablation. The figures and data within sFig. 1 offer a detailed visual representation of the time course and various remodeling patterns observed across five distinct animal experiments (Mice A-E). These patterns include collateral outward remodeling, reopening of arterial and venous segments, and instances of permanent segment occlusion. The selection of Mouse E as a representative case for in-depth analysis in sFig. 1 serves to illustrate consistent changes seen across all mice while supplying essential anatomical data for subsequent biological and mathematical modeling endeavors. sTable 6 complements this by summarizing the observed remodeling patterns at different time points, highlighting the persistence of both outward and inward remodeling in arterial and venous segments throughout the observation period. Additionally, the findings emphasize the network’s remarkable adaptability, with the ability to achieve permanent occlusion in some vessels while also demonstrating the capacity for gradual reopening over time in others. Together, sFig. 1 and sTable 6 could offer critical insights that have relevance for both experimental investigations and potential clinical applications.

The mouse shown on row E in sFig. 1 was found to be representative of the remodeling processes and was used for more detailed analysis. Extensive flow redirection and vascular remodeling occurred after ablation in the arterial side of the vasculature (Fig. 2). The pattern of upstream and downstream remodeling of the network vessels is consistent with previous results in the mouse gracilis artery^4^. Interestingly, veins remodeled more than arteries, but with no observable change in flow direction.

The process of vessel regeneration via angiogenesis was consistently observed on the arterial side of the vasculature. There was directed, collective migration of endothelial and smooth muscle cells from the damaged segments that eventually spanned the ablation sites and reconnected the damaged arteries. In one case, the ablated artery segment was in the process of reconnection at the termination of our observations (Fig 3A).

On the venous side, flow was often rerouted locally around the ablation sites through small postcapillary venules that were not readily observable prior to the ablation, which subsequently increased dramatically in diameter to re-establish the original vein diameter. This rerouting depended on the pressure drop across the ablation site: if the ablation interrupted flow in a large vein, rerouting and subsequent vessel remodeling occurred to compensate for the ablation (sVideos 1A-D, 3A-D). Although some of the small vessels that constituted these new, remodeled pathways could be identified in the pre-ablation images, it is not clear whether angiogenesis was involved in making some of the rerouting connections to the vein. Interestingly, if there was little pressure drop across the ablation site, the re-routing was not apparent. Veins that were ablated at two locations along the length resulted in a downstream interruption with high trans-ablation pressure drop, but also an upstream ablation with low pressure drop (because flow in the vessel had already been blocked by the downstream ablation). In this case, there was re-routing around the downstream ablation, but not around the upstream ablation (sFig. 1A). Conversely, there was no reconnection of the downstream vessel via angiogenesis, but there was for the upstream segment.

The study has several notable limitations. First, we applied multiple laser ablations in each network rather than single ablations. Systematic analysis of the effects of individual ablations is challenging because the network in each DSFC is unique, so identifying equivalent vessels in different mice is difficult. In addition, in our experience, ablating only one vessel results in little perturbation in flow over the entire network. For this reason, multiple ablations were more appropriate to analyze flow redistribution. In addition, some aspects of this study focused on one specific case study (Mouse E), and the findings may not be fully generalizable. However, we observed qualitatively similar adaptation processes in Mice A-D (sFig. 1). Such common remodeling patterns included venous and arterial collateral development and segment reopening, and both outward and inward remodeling, with variations in the time frames for each mouse. We should also reiterate that our imaging was not, in general, able to resolve all the vessels in the DSFC, and there were many capillaries and microvessels that were not visible or quantified.

Network connectivity plays a major role in flow patterns and vascular remodeling. Flow reversals are unequivocal evidence of redundancy, indicating that a region of tissue can be reached by more than one pathway. Because they can arise from variable demand, they can maintain distal vessels with flow reversal and probably shear stress adaptation (not addressed in the present work). The vessels showing the highest degree of uncertain flow direction (yellow and red segments in Figs. 8 and 9) seem to be most likely to undergo flow reversal after others are ablated in the arterial network. These segments were also more likely to increase in diameter to accommodate the change in flow. Nonetheless, due probably to the large density of vessels in the network, the vessels with reversed flow did not remodel significantly as observed previously in the gracilis artery of the mouse^2^. Several vessels at the periphery changed direction, but they did not experience a large, visible increase in diameter, which could mean they have changed direction of flow before or that change in the direction in flow by itself does not increase the shear stress and diameter significantly. Regardless, a change in direction of flow was necessary to restore tissue perfusion and maintain pressure in peripheral vessels, which were observed to maintain flow and had little changes in diameter. While full validation of the model awaits more rigorous testing with a larger data set, our present study demonstrates concepts of vascular redundancy and remodeling that are relevant to other microvascular networks with collateral vessels such as the brain, retina, and coronary circulation. Furthermore, the experimental and modeling approaches described here could be used to estimate the remodeling potential of any vascular network under changing blood flow conditions.

## METHODS

### Experimental model

#### Animal models.

The protocol for the animal experiments was reviewed and approved by the Institutional Animal Care and Use Committee of the Massachusetts General Hospital. The procedures were performed in accordance with the approved guidelines. The reporting in the manuscript follows the recommendations in the ARRIVE guidelines. We have generated αSMA^+^-DsRed/Tie2^+^-GFP/FVB double transgenic mice line by crossing the Tie2^+^-GFP/FVB mice^24^ with the αSMA DsRed mice^25^. Once established, this transgenic line has been backcrossed to FVB mice at least 10 generations^25–27^.

#### Dorsal skin fold chamber (DSFC).

A DSFC was implanted in ten mice as described before^28^ (Fig. 1). Briefly the animals were anesthetized via intraperitoneal K&X injection. The back fur was removed over a 2.5×2.5cm area with an electric shaver and topical hair removal lotion. The skin is lifted, and the chamber is fixed to the skin with metal screws. A circular skin area of 1cm in diameter is removed to install the glass slide. The chamber was maintained throughout the duration of the experiment. The glass coverslip was not removed during the laser ablation and imaging procedures. The initial set of five mice was employed to refine the experimental and imaging techniques (data not presented). The subsequent group of five mice, identified as Mice A-E (refer to sFig. 1 and sTables 1–6), was included in the present study. These mice were assessed at different time intervals following diverse levels of laser ablation to determine general remodeling patterns. Mouse E, selected as a representative case, is thoroughly discussed to exemplify consistent changes observed in all mice and to furnish anatomical data for the mathematical model. The diameter data presented in sTables 1–5 are expressed as mean values along with their respective standard deviations, for three equidistant radial regions: proximal, medial, and distal. To define these regions, we calculated the distance from each arterial/venous vessel fragment to the nearest corresponding ablation site. Subsequently, all these distances were sorted in ascending order, and the maximum distance was divided into three equal parts, with segments falling into each third reported accordingly. sTable 6 was constructed based on the observations presented in sFig. 1, where “Y” indicates the occurrence of a specific remodeling pattern, and “N” indicates the absence of such a pattern.

#### Multiphoton laser imaging and ablation.

A modified in-house multi-photon laser^29^ was used for the laser ablation and imaging. The laser beam was programmed to scan a single line oriented perpendicular to the vessel longitudinal axis, with the length of approximately 1.5x the vessel diameter. The laser power was increased to 1W. Several line scans were run side to side across the vessel until the vessel wall constricted and then disrupted, leading to blood stasis (evaluated via light microscopy). Subsequent imaging of the vessel was performed with the same MPLSM after returning to normal laser power. A Nikon SMZ 2500 stereomicroscope (Nikon Instruments Inc., Malville, NY) was used to image the microvascular structure *in vivo* throughout the remodeling process.

#### OCT imaging.

A custom-build OCT system^30^ was used for angiographic and quantitative flow imaging. Angiographic methods follow those described before^30^. Quantitative flow information was derived from the OCT amplitude-decorrelation rate^31–33^. Briefly, the temporal rate of the OCT amplitude signal decorrelation was measured at each voxel and used as an indication of relative flow speed. The OCT data were not used to draw conclusions on absolute flow speed. Due to gradient effects^32,34^, measurements in vessels with diameters comparable to or slightly larger than the imaging resolution (~15 μm) were not used in the analysis.

### Computational vascular network model

#### Microvascular network fluid dynamics.

The vascular network data (number of vessels, connectivity matrix, vessel diameters and lengths) were measured from digital images of the microvascular network in all visible segments using the NIH ImageJ software. Arteries and veins were identified by morphology, location and flow direction from live observation and movies. Arteries and veins are usually paired, and the artery has a smaller diameter. Smaller arterioles and venules were identified by tracing them back to the larger arteries and veins. The map of the vascular network was extracted from low magnification images presented in Figs. 1A and B and Fig. 2. The diameter measurements were performed at a higher resolution (e.g. Fig. 3A and sFig. 2) which allowed clear visualization of the inner vessel diameter of viable blood vessels as marked by the presence of the blood in their lumen. The measurements were performed at the same magnification at all time points. The statistical analysis including the number of total vessels of a given diameter and % diameter changes as well as the graphical representation of the data were performed in Microsoft Excel and GraphPad Prism version 9.5.0 for macOS, GraphPad Software, San Diego, California USA, www.graphpad.com. The mathematical model for calculating the hemodynamic parameters (blood flow, intravascular pressure, resistance) was described before in detail^1,4^. Whereas calculations of flow and pressure were previously used to predict adaptation in the vessel diameters^1,4^, here we assume fixed diameters, but seek the sensitivity of flow in each vessel to distant changes in the network topology. The present model aims to provide insight into the sensitivity of flow to distant changes in the network topology rather than to predict adaptation due to shear stress and pressure as was done before^1,4^.

Briefly, the vessel segments were represented as cylinders with bifurcation nodes between them. Assuming steady state laminar flow of a Newtonian fluid, the flow rate for the segment connecting nodes i and j is given by $Q_{ij}=\frac{\left(P_{i}-P_{j}\right)}{R_{ij}}$, where $R_{ij}=128L_{ij}\mu /\pi D_{ij}^{4}$ is the flow resistance of segment ij, and $L_{ij}$, and and $D_{ij}$ are its length and diameter, μ=3cP is viscosity. Conservation of mass at node i requires that:

$$\frac{\left(P_{i}\;-\;P_{v}\right)}{R_{TA}}+\sum_{j=\;connecting\;vessels}\;\frac{\left(P_{i}\;-\;P_{j}\right)}{R_{ij}}=0$$

where $R_{TA}$ is the resistance of the terminal arteries relative to the venous network which is assumed to be at a constant pressure $P_{v}$. The resulting system of linear equations can be solved for the $P_{i}$’s and the flows in each segment provided that all of the pressures at the boundaries of the field of view (FOV) are known. Here we assume a known pressure for the largest vessel entering the FOV. The remaining smaller vessels that cross the boundary have pressures that are unknown but can be estimated from a simulated annealing method (SAM) that seeks the optimal fit between the experimentally observed flow directions in all segments within the FOV and the directions obtained from our estimated boundary pressures.

#### Simulated annealing method.

The theoretical basis of the simulated annealing method was described before^35^. To implement this method, we make a random assumption/guess of the unknown boundary pressures, and then calculate the flow directions throughout the network based on this guess. By iteratively adjusting the guessed boundary pressures, the SAM seeks a global minimum for an error function (E) equal to the total number of segments with calculated flow directions that differ from those experimentally observed. The error function can be unweighted, weighted by the estimated flow or diameter, or both. The SAM usually accepts a better guess but avoids getting trapped in local minima by occasionally accepting a less good estimate with a probability proportional to exp[−(E(n)−E(n−1))/T], where n is the annealing iteration number and T is a gradually decaying pseudo temperature. The boundary pressures are updated 5,000 times for each set of initial guesses until the error function reaches a stable minimum.

The entire SAM optimization process is repeated 100 times with a new, random set of initial pressures for each run yielding a set of boundary pressures that minimizes the number of incorrectly predicted flow directions. Because the optimization process is stochastic, the variability in segment flow rate between optimization trials serves as a measure of how sensitive flow in that segment is to changes elsewhere in the network such as those imposed by vessel ablation.

## Data Availability

The datasets and computer code generated during and/or analyzed during the current study are available from the corresponding authors on reasonable request.
